# Effect of Cocoa Products and Its Polyphenolic Constituents on Exercise Performance and Exercise-Induced Muscle Damage and Inflammation: A Review of Clinical Trials

**DOI:** 10.3390/nu11071471

**Published:** 2019-06-28

**Authors:** Marika Massaro, Egeria Scoditti, Maria Annunziata Carluccio, Antonia Kaltsatou, Antonio Cicchella

**Affiliations:** 1 National Research Council—Institute of Clinical Physiology, Laboratory of Nutrigenomic and Vascular Biology, 73100 Lecce, Italy; 2 FAME Laboratory, Department of Physical Education and Sport Science, University of Thessaly, 42100 Trikala, Greece; 3 Department for Quality of Life Studies, University of Bologna, 40126 Bologna, Italy

**Keywords:** athlete, cocoa, chocolate, exercise performance, oxidative stress, performance, physical exercise, polyphenol, skeletal muscle, inflammation

## Abstract

In recent years, the consumption of chocolate and, in particular, dark chocolate has been “rehabilitated” due to its high content of cocoa antioxidant polyphenols. Although it is recognized that regular exercise improves energy metabolism and muscle performance, excessive or unaccustomed exercise may induce cell damage and impair muscle function by triggering oxidative stress and tissue inflammation. The aim of this review was to revise the available data from literature on the effects of cocoa polyphenols on exercise-associated tissue damage and impairment of exercise performance. To this aim, PubMed and Web of Science databases were searched with the following keywords: “intervention studies”, “cocoa polyphenols”, “exercise training”, “inflammation”, “oxidative stress”, and “exercise performance”. We selected thirteen randomized clinical trials on cocoa ingestion that involved a total of 200 well-trained athletes. The retrieved data indicate that acute, sub-chronic, and chronic cocoa polyphenol intake may reduce exercise-induced oxidative stress but not inflammation, while mixed results are observed in terms of exercise performance and recovery. The interpretation of available results on the anti-oxidative and anti-inflammatory activities of cocoa polyphenols remains questionable, likely due to the variety of physiological networks involved. Further experimental studies are mandatory to clarify the role of cocoa polyphenol supplementation in exercise-mediated inflammation.

## 1. Introduction

Skeletal muscle exerts a dominant role in postural control, the protection of internal organs, locomotion, and other physiological functions requiring energy-mediated mechanical activity based on muscle fiber contraction [1]. Since skeletal muscle is the largest organ in the body [2] and acts as a thermal machine, skeletal muscle energy consumption plays a key role in the regulation of whole-body energy homeostasis [1]. However, recently, skeletal muscle has been proposed as a potential “endocrine organ” that is able to orchestrate the release of an array of muscle-derived signaling molecules or myokines, including interleukin(IL)-6, IL-7, IL-8, IL-10, IL-15, and IL-1 receptor antagonists, irisin, and myostatin [3]. The tightly regulated release of such myokines is involved in many exercise-induced metabolic adaptations, such as those related to glycemic control and lipid homeostasis [4], as well as in the regulation of muscle fiber composition and contractility [5]. Furthermore, the increases in muscle mass and vascularization appear to be regulated by myokines [5]

From a pathogenic point of view, it is well known that physical inactivity predisposes individuals to developing a variety of chronic diseases [6], while the practice of regular physical exercise exerts a range of beneficial effects on body health [7]. Regular exercise is indeed recognized to improve whole-body energy metabolism and to make muscles stronger and more resistant to fatigue [8]. However, this relationship can be described by an inverted U-shaped dose–response curve (exercise hormesis theory [9]), as follows: when the stressor (the exercise bout) is absent, no adaptation occurs. Positive, healthful adaptation starts when the dose of exercise is within a specific intensity and duration range and is followed by a rest period [10]. However, if exercise bouts are too heavy or extended, as in athletes involved in prolonged and intensive exercise activities [11], or are not followed by rest periods, muscle fiber damage and inflammation may occur, requiring exogenous intervention to re-establish normal muscle functionality [10] or to delay the onset of fatigue through substances acting on the central nervous system (CNS) [12]. Mechanical and metabolic stress accompanying excessive exercise may result in damage to the integrity of myofibers and may cause non-permanent reductions in contractile function [13]. At the same time, muscle injury triggers a complex tissue inflammatory reaction featured by the release of pro-inflammatory myokines and stress hormones with the final aim of repairing damaged muscle and orchestrating regenerative and adaptive processes [14]. Under these conditions, the activation of resident endothelial cells and the recruitment of inflammatory leukocytes, including neutrophils and macrophages, occurs, leading to cumulative production of pro-inflammatory and anti-inflammatory cytokines that, together with myokines, actively contribute to the removal of necrotic tissue and cellular debris [10]. Once necrotic tissue is removed, satellite cells proliferate to regenerate muscle tissue [15]. By this multi-step process, exercise-induced inflammation supports cellular remodeling, promotes hypertrophic adaptations, and restores tissue homeostasis and contractile function [16]. 

Besides muscle glycogen breakdown and increased levels of calcium, catecholamines, growth hormone, and cortisol [17], cytokine expression and release during exercise is mediated by the overproduction of reactive oxygen species (ROS) [18]. Exercise may indeed increase oxygen consumption (VO_2_) up to 20 times above resting values [18]. In the mitochondria of muscle cells, this translates to 200-fold greater oxygen utilization and the subsequent production of a large amount of ROS [18]. ROS generated through this route may lead to oxidative damage to the mitochondria and muscle contractile proteins, with subsequent direct induction of muscle damage and fatigue after exercise [10]. Furthermore, ROS also orchestrate the activation of redox-sensitive signal pathways that control cytokine production and muscle adaptation, such as those involved in the activation of nuclear factor(NF)-κB, Nuclear Factor of Activated T-cells (NFAT), Nuclear factor erythroid 2-related factor(Nrf2) and heat shock proteins (HSPs), and Peroxisome Proliferator-Activated Receptor-gamma Coactivator (PGC)-1α [17]. In particular, the expression of most immune-related genes requires activation of the pleiotropic, redox-sensitive NF-κB and its binding to specific consensus sequences to activate gene transcription [19]. Among these genes, there are many myokines including IL-1, IL-8, IL-6, chemotactic factors (such as the monocyte chemotactic protein (MCP)-1, many endothelial leukocytes, adhesion molecules (including the vascular cell adhesion molecule (VCAM)-1), and the pro-inflammatory prostaglandin producing enzyme cyclooxygenase (COX)-2 [20]. In this way, NF-κB promotes the release of pro-inflammatory mediators, including chemokines, cytokines, acute phase proteins, and adhesion molecules, to facilitate the regenerative response in damaged skeletal muscle [21]. Furthermore, several studies have clearly indicated that muscle ROS and NF-κB also activate other important cell signaling pathways, leading to skeletal muscle adaptations to exercise such as mitochondrial biogenesis [22,23] and endogenous antioxidant defense [24].

However, due to overwhelming ROS production and the overcoming of antioxidant tissue defense during periods of intensified physical training, chronically activated NF-κB and the following dysregulated production of inflammatory myokines may lead to skeletal muscle atrophy and soreness [20]. 

Prolonged, high-intensity, strenuous or unaccustomed bouts of exercise have been experimentally associated with increases in contractile-induced damage and inflammation reactions in skeletal muscle [25]. For example, dysregulation in the inflammatory system has been observed in athletes undergoing intense periods of physical training, as highlighted by excessive delayed-onset muscle soreness, muscle stiffness, a reduction in muscle strength, increased creatine kinase (CK) activity, and impaired immune function [25]. Under these conditions, the functional performance of skeletal muscle has been shown to be reduced for at least 24–96 h [26], with serious health and economic implications for professional athletes and related clubs and societies [27]. Therefore, while exercise-induced inflammation is necessary for muscle repair and adaptation, the uncontrolled proliferation of inflammatory cells and oxidants can exacerbate muscle damage and impair muscle function. 

The growing evidence on exercise-induced oxidative damage and impairment of athlete performance has spurred intense research on the evaluation of muscle protection by antioxidant supplementation in exercising individuals [25]. Although many studies have shown potential therapeutic effects by antioxidant supplementation [28,29,30], results from several others remain inconsistent. For example, supplementation with vitamin C and N-acetylcysteine was found to increase oxidative stress and cell damage following eccentric exercise [31]. Similarly, in a recent study by Bailey et.al, a combination of vitamins C and E was shown to increase markers of oxidative stress and inflammation [32]. Since low amounts of free radicals may act as cellular signals to enhance antioxidant defenses, rather than being deleterious, as occurs at higher concentrations, it has been suggested that high antioxidant supplementation may attenuate some of the exercise-induced cellular signals that stimulate adaptations in skeletal muscle [18]. This has been proven to be true upon oral supplementation with vitamins C and E [22,33] after an endurance trial and after high intensity exercise. 

Based on these findings, dietary recommendations in exercising individuals should emphasize the consumption of a well-balanced diet and/or natural antioxidant-rich foods such as cocoa and chocolate, rather than taking antioxidant supplements. This “nutraceutical strategy” has been increasingly proposed as a potential suitable tool for preventing or reducing oxidative stress and related inflammation during intensive physical training. In particular, besides being a high energy-dense foods, cocoa and cocoa products including chocolate are a rich source of antioxidant polyphenols that have proven to possess health-promoting effects through their antioxidant, anti-inflammatory and metabolic properties [34].

The overall goal of the current review was therefore to examine the effects of cocoa polyphenols and on different redox-related outcome parameters, including oxidative and inflammatory biomarkers, and muscle performance and recovery.

## 2. Polyphenol Antioxidant Profile in Cocoa and Chocolate

Chocolate and cocoa are foodstuffs derived from the beans of the Theobromacacao tree [35]. After harvesting, cacao pods are processed to form a liquid paste known as “cocoa liquor”, the cocoa component is richer in healthful bioactives [35]. The healthiest types of chocolate are indeed those containing higher amounts of cocoa liquor, namely dark chocolate, and, to a lesser extent, milk chocolate [36]. Most of the health effects of cocoa-rich chocolate are due to the high content of nutritional polyphenols in cocoa liquor [37]. In the last thirty years, polyphenols have attracted much interest owing to their antioxidant capacity (free radical scavenging and metal chelating ability) and their possible beneficial implications in human health, such as in the treatment and prevention of cancer and cardiovascular disease [38]. Hence, cocoa has the health effects generally ascribed to polyphenol consumption [34]. 

**Figure 1:**
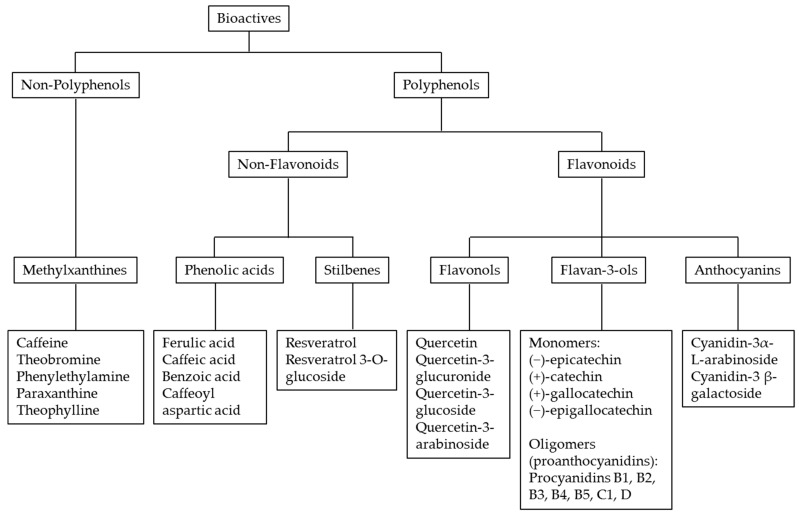
Main bioactive compounds of cocoa and chocolate.

Cocoa seeds contain many bioactive compounds including high levels of polyphenols (12–18% of dry weight) as well as fatty acids, vitamins, minerals, fiber, and several methylxanthine alkaloids (4% of dry weight), which are psychoactive dopaminergic substances such as caffeine, theobromine, theophylline, phenylethylamine, and paraxanthine (Figure 1). 

From a bromatological point of view, the most abundant polyphenolic classes in cocoa and chocolate are:

(a) the monomeric flavan-3-ols or catechins (up to 29–38% of total cocoa polyphenols) including catechin, gallocatechin, and epigallocatechin (Figure 1). In particular, epicatechin may represent up to 35% of total polyphenols in both cocoa powder and chocolate [39]; 

(b) the proanthocyanidins (up to 58–65% of total cocoa polyphenols), which are polymers of epicatechin and catechin and include mainly dimeric and trimeric molecules [39]; 

(c) the anthocyanins (up to 4% of total cocoa polyphenols), which include cyanidin-3α-L-arabinoside and cyanidin-3 β-galactoside as the most represented compounds [39].

Finally, some other phenolic compounds can be found at very low concentrations including phenolic acids, caffeoyl-conjugates, and stilbenes [39]. Notably, the proportions of these compounds depend on the cultivar, origin, agricultural practices, and postharvest practices and processing. In particular, the phenolic content and profile of cocoa-derived products change considerably during the manufacturing process, especially during fermentation and alkalization [40].

The metabolic fate of phenolic compounds after ingestion is a critical aspect in determining the health effects of such compounds and the mechanisms through which they exert their biological activities. While oligomers larger than trimers are unlikely to be absorbed, dimers have been reported in human plasma after cocoa ingestion [41]. Catechin and epicatechin are well absorbed in the small intestine. They are transiently detectable in plasma as glucuronide-conjugated metabolites and sulfate groups with a maximum plasma concentration at around 2 h and a return to baseline values within 24 h (in general, in 6–8 h) of the consumption of a flavonoid-rich chocolate meal (such as dark chocolate, dose range 40–100 g) [42,43,44]. Recently, a fine metabolomic assessment on human plasma and urine samples collected at 2 and 6 h after the consumption of flavan-3-ol-enriched dark chocolate highlighted the presence of discriminant epicatechin derivatives in the urine, thus confirming the high bioavailability of cocoa flavanols [45]. Also, in athletes, the assessment of the plasma epicatechin concentration is often used as a biomarker of chocolate intake to correlate the effects of chocolate on metabolic endpoints to the effective polyphenol absorption [46]. However, to the best of our knowledge, no study has specifically addressed the metabolism and pharmacokinetics of chocolate polyphenols in athletes following physical exercise.

## 3. Literature Search Strategy

Two electronic databases were consulted: PubMed and Web of Science. Key terms that were included and combined were “intervention studies”, “cocoa polyphenols”, “exercise training”, “inflammation”, “oxidative stress”, and “exercise performance”. The final search was carried out on 24 April 2019. The search strategies were combined, and duplicates were removed by Endnote X7 (Clarivate Analytics, previously Thomson Reuters, Philadelphia, PA, US) and manually. Studies in this section needed to fulfill the following inclusion criteria: (i) research conducted with human participants and (ii) original data from randomized clinical trials (RCTs) on cocoa ingestion with an acute or long-term exercise intervention. The exclusion criteria were (i) studies written in languages other than English, (ii) animal or in vitro studies, (iii) congress or workshop publications, (iv) studies in which no exercise was performed, (v) studies in which no supplementation was given, and (vi) studies in which mixed supplements were given. No limits were used concerning the year of publication. The inclusion or exclusion of articles was determined by applying the above criteria on the title and abstract as a first screening and on full texts as a second screening. Case studies and reviews were excluded, although the respective references were consulted and integrated into this revision if responding to the above-mentioned criteria.

## 4. Effects of Cocoa Polyphenols on Exercise-Induced Oxidative Stress, Inflammation, and Recovery 

Thirteen intervention studies with parallel or crossover design met all inclusion criteria and were included in the analysis. Overall, they involved a total of 200 participants, mostly well-trained athletes, and examined the effects of cocoa polyphenol intake on exercise-induced changes in plasma markers of inflammation, oxidative stress, and performance. 

Eight studies administered polyphenols in the form of solid chocolate [46,47,48,49,50,51,52], while five studies examined the effects of cocoa polyphenols in a liquid formulation [53,54,55,56,57]. The number of polyphenols tested was rather varied, ranging from 36 to 1000 mg, administered in an acute (2 h before exercise), sub-chronic (for 2 weeks before exercise), and chronic (up to three months before exercise) fashion. No study involved female athletes, and tested sports and exercise disciplines were mostly cycling, football, and running. The results are compared and summarized in Table 1 for acute studies, and Table 2 for sub-chronic and chronic studies. 

The selected studies examined the effects of cocoa polyphenol supplementation on the exercise-mediated deregulation of oxidative redox status by evaluating markers of oxidative damage (including malondialdehyde [MDA], 4-hydroxynonenal [4HNE], F2-isoprostane, and thiobarbituric acid reactive substances [TBARS], and protein carbonylation) and antioxidant protection (glutathione and total antioxidant status—TAS) [46,47,48,50,52,53,54,55,57,58]. Three studies analyzed the effects of the acute administration of cocoa polyphenols on exercise-induced oxidative stress (Table 1). In particular, Davison et al. observed that the consumption of a single low amount (248 mg) of cocoa flavanols as solid dark chocolate 2 h before exercise was able to enhance pre-exercise antioxidant status and, correspondently, tended to reduce the post-exercise plasma F2-isoprostane content as compared with a control condition, thus suggesting an improvement in antioxidant defense for athletes following the consumption of dark chocolate enriched in polyphenols [58]. Similarly, Wiswedel et al. observed that a single low intake (186 mg) of cocoa flavanols did not increase the total antioxidant capacity but was able to prevent increases in F2-isoprostane and MDA induced by 30 min of cycling [57]. On the other hand, Decroix at al. evaluated the consumption of a single but higher dose (900 mg) of cocoa flavanols in the form of a beverage given 30 min before exercise. Again, the total antioxidant capacity was significantly increased by cocoa but without a clear effect in terms of MDA [54]. Furthermore, Davidson et al. [58] and Decroix et al. [54] also evaluated the effect of polyphenol ingestion on a classical marker of inflammation, IL-6, and specifically evaluated whether cocoa supplementation affects exercise performance and recovery. Both authors independently observed that cocoa flavanols had no effects on IL-6 plasma levels or on exercise performance and recovery. In agreement with Davidson [58] and Decroix [54], Stellingwerff et al. [51] and Peschek et al. [56] did not find any improvement in physical performance following the acute administration of low or medium concentrations of cocoa polyphenols following downhill running [56] or cycling [51] (Table 1). 

Seven studies instead examined the effects of sub-chronic administration (from 5 days up to 2 weeks) of cocoa flavanols before or during exercise (Table 2). Allgrove et al. [47], Patel et al. [49], and Singh et al. [50] evaluated the effects of low amounts of cocoa polyphenols (about 200 mg/day) on cycling-mediated oxidative stress and inflammation. After two weeks of supplementation plus a double dose of cocoa polyphenols on the testing day, 2 h before cycling exercise for 1.5 h, Allgrove et al. [47] found a significant increase in the total antioxidant activity capacity but no effects on exercise performance or on the plasma levels of several pro-inflammatory myokines, including IL-6, IL-1RA, and IL-10, were evident. However, under similar experimental conditions (in terms of polyphenol concentration and loading time), Patel et al. observed an improvement in muscle physical performance as indicated by a lower oxygen demand during analogous moderate intensity exercise [49]. Finally, Singh evaluated the effects of a polyphenol loading time of 7 days on trained and untrained cyclists [50]. However, he failed to show improvements in the total antioxidant status in both sub-types of athletes [50]. Slightly different were the effects shown for sub-chronic cocoa polyphenol supplementations in well-trained football players. Under these conditions, both Fraga et al. [48] and Gonzalez-Garrido et al. [55] showed significant improvements in antioxidant defense and, interestingly, in exercise performance following low and high polyphenol administration, respectively [48,55]. However, these positive results are not supported by the recent findings of de Carvalho et al. in rugby players [53]. Here, the sub-chronic administration of cocoa polyphenols neither improved oxidative stress markers and plasma CK levels, nor enhanced athletic performance [53]. Only two studies have examined the effects of the very long chronic administration (three months) of cocoa polyphenols on oxidative stress and inflammation markers in football players [46] and cyclists [52] (Table 2). Both studies concordantly observed improvements in different oxidative stress markers including the hydrogen peroxide breakdown activity level, reduced glutathione, and protein carbonyls [46,52]. However, discordant effects were observed for exercise performance outcomes 

## 5. Discussion

In this review, we examined the effects of cocoa polyphenol intake on exercise-mediated oxidative stress and inflammation, performance, and recovery. 

Examining thirteen studies, we observed that acute, sub-chronic, and chronic cocoa polyphenol intakes reduce exercise-induced oxidative stress but not inflammatory markers, while the effects on exercise performance and recovery remain controversial. 

Both acute and long-term exercise are now widely recognized as potential pro-oxidative and pro-inflammatory promoters [25]. Although the generation of ROS represents a physiological process in most human metabolic reactions, when ROS production and related endogenous antioxidant abilities are imbalanced, a maladaptive biological response may occur, leading to both oxidative stress and inflammation [59]. In muscle cells, aerobic energy production generates a significant amount of ROS, which can increase by up to 10–20-fold during physical exercise [60]. Although moderate levels of ROS may serve as signaling molecules that mediate muscle repair and adaptation, protein turn-over, mitochondrial biogenesis, and the upregulation of antioxidant enzyme levels [9], increasing evidence suggests that high and unbalanced ROS levels are able to deregulate the redox state and induce a high rate of pro-inflammatory myokine release in muscles, which may lead to contractile muscle dysfunction, accelerated muscle fatigue, longer recovery time, and reduced exercise performance [61].

Therefore, although inflammation provoked by physical activity is directly related to the intensity of the effort, if the exercise bout is excessive in terms of intensity and/or duration, the human body may need some kind of external “aid” to activate the recovery process. A list of forbidden substances is issued and continuously updated by the anti-doping agency [62,63], leaving many possibilities open for the introduction and use of non-doping supplements. A number of substances of vegetal origin have been proposed as potential tools to delay fatigue onset during physical activity and/or to promote the recovery process. 

Chocolate and cocoa polyphenols are among these substances. Different from other proposed food supplements, chocolate, and in particular dark chocolate, has been postulated to possess a dual function: it might act as an ergogenic support due to its characteristic richness in saturated fat and sugar, but being also rich in methylxanthines (including theobromine, and caffeine), it might directly act as a stimulant on the CNS [38]. Furthermore, due to its richness in antioxidant polyphenols, it might impact exercise-mediated redox deregulation and inflammation. Evidence on the effect of chocolate on brain functions has been reported, especially in terms of delayed perception of fatigue [12]. Some positive evidence exists on the influence of the consumption of a chocolate milk beverage between three bouts of intense exercise in cycling [64] and after endurance sport in general [65]. Subsequent research failed to demonstrate the effectiveness of chocolate milk in comparison with raw milk plus honey in reducing delayed muscle soreness [66], making evidence of the utility of chocolate in exercise recovery weak. In these studies, however, the chocolate polyphenol content was very low or absent and/or not specifically addressed, thus introducing a bias in the interpretation of results. 

The data reviewed here are clearly indicative of a dual effect by cocoa polyphenols. On the one hand, in a rather homogeneous fashion, they collectively highlight the ability of cocoa polyphenols to exert antioxidant effects, as indicated by reduced accumulation of lipid and protein oxidation products and by the strengthening of the athlete antioxidant capacities. On the other hand, the data do not show a clear improvement in post-exercise pro-inflammatory status, and the evidence is rather faint regarding the effect of cocoa polyphenols on exercise performance and post-exercise recovery. 

Therefore, although the protective effects of polyphenol supplementation have been largely documented in humans as well as in animal and in vitro studies [67], the available data reported here show contradictory results and, in agreement with other revisions [68,69,70], do not sustain a clear benefit of cocoa polyphenol supplementation in ameliorating the inflammatory changes induced by exercise. 

Notably, most studies are not directly comparable because they used low-flavanol cocoa or milk chocolate as controls, with different contents of not only flavanols but also other potential bioactives including methylxanthines. Moreover, potential interactions between methylxanthines and cocoa polyphenols have been found, resulting in synergistic/additive effects and increased bioavailability of flavanols when co-ingested with methylxanthines [71]. It seems, therefore, appropriate to match the test and control treatments for methylxanthine content to separate the effects of cocoa polyphenols. This aspect may in part contribute to some inconsistencies in studies’ results. However, among the selected papers, Stellingwerff et al., even observing an increase in theobromine and epicatechin plasma level after dark chocolate consumption, did not find improvement in exercise performance [51], this suggesting the necessity of further investigations. 

Many other factors may contribute to explaining these disappointing results. First of all, previous positive results mainly come from studies investigating polyphenol activity in pathological conditions (in particular cardiovascular disease and cancer) where the redox status is highly unbalanced and the addition of dietary antioxidants may effectively favor the restoration of the right equilibrium [72,73]. On the other hand, finely tuned ROS production during exercise is essential to promote the expression of several proteins that are crucial for exercise-induced adaptation, and the use of antioxidants in supra-physiological doses may be detrimental and/or alter the oxidative stress response in terms of pro-inflammatory gene induction. Thus, antioxidant supplementation might produce adverse consequences by decreasing the ROS concentration beyond the required homeostatic level. Finally, the additional effects of exogenous supplemental antioxidants on different types of exercise are difficult to predict, because exercise itself is a positive stimulus that generally drives the antioxidant capacity. Exercise itself is a strong masking agent that is able to obscure any possible effects of a single substance [9].

It is also important to consider different biological responses related to the type of exercise and, also, that antioxidants might be effective during specific periods of training and that the requirements may vary according to different seasonal needs [74]. For all of these reasons, a personalized plan that considers all of the specific requirements of athletes during the different phases of training would represent the best option to improve global performance, since the training process is highly variable and dependent on a wide range of factors [74]. All of these management aspects should be taken into consideration when planning future experimental studies to ascertain the protective role of cocoa polyphenols in professional and amateur athletes. 

Finally, between-studies comparisons are sometimes difficult because of the often-limited sample size, as well as the different populations, types of training and physical activity levels, and background diets of the participants that may influence the effects of chocolate supplementation and exercise. In general, selected studies recruited no more than 10–15 subjects, with the exception of only three studies [46,47,48] that recruited 20 or more subjects. A small sample size reduces the power of the study and increases the margin of error, rendering the study meaningless. Relatively more homogeneous conditions were observed under acute cocoa administration. Here, the most recurrent kind of physical exercise was cycling but practiced for different times ranging from 2.5 h [51,58] to 30 min [54,57].

Levels of serum polyphenol concentrations, as an index of adherence to the protocol, were evaluated in all acute studies [51,54,57,58], except in the study of Peschek [56]. Data on cocoa polyphenols availability suggest a more frequent increase in epicatechin levels [51,54,58] with respect to catechin [57]. On the other hand, under sub-chronic and chronic cocoa administration, the assessed physical activities were more diversified including football [46,48,55] and rugby [53] besides cycling [47,49,50,52]. Moreover, levels of serum polyphenol concentrations were evaluated in only two studies with discordant results since, under the same sport activity, i.e., the football, Fraga et al. did not find any increase in the plasma levels of epicatechin and catechin [48], while Caravetta et al. did find a clear increase in the epicatechin levels [46]. However, independent of the increase in the plasma levels of cocoa polyphenols, both studies observed a clear improvement in the oxidative stress status.

It is conceivable that the effects of cocoa flavonoids, as well as of other food supplements, may be more evident as the severity and temporal extension of inflammation increases, which mostly results from exhausting low-intensity aerobic exercise. So, the standardization of physical activity intervention, sample size, and administered supplement should be carefully addressed in further studies concerning cocoa flavonoid usage in professional and amateur sport.

Few studies have tried to deepen the understanding of the mechanisms of polyphenol activity by examining the activation of pro-inflammatory transcriptional factors through muscle biopsies before and after antioxidant supplementation and training [75,76]. Nieman et al. obtained disappointing results regarding muscle NF-κB activation, which was shown to be unaffected by exercise and following quercetin supplementation [11]. Such mechanistic exploration should be reproduced and confirmed. Finally, only four studies [47,54,55,58] evaluated the plasma levels of pro-inflammatory markers, including IL-6, IL-1, TNF-α, IL-1 RA, and CRP. It is possible that the tested inflammatory biomarkers are not sensitive or specific enough to be modulated through the cocoa-mediated nutraceutical approaches. The array of myokines tested should be broadened in the search for potential more sensitive and specific biomarkers.

In conclusion, the evidence supporting the effects of the consumption of cacao or dark chocolate on exercise performance and/or exercise-mediated inflammation remains weak.

At present, there is no evidence supporting the use of cacao or dark chocolate as an ergogenic aid. Evidence on the antioxidative and anti-inflammatory effects of cocoa polyphenols in athletes remains weak due to the variety of physiological networks involved. Further experimental studies are necessary in order to clarify the interaction of exercise training and cocoa antioxidant supplementation and the beneficial effects of cocoa polyphenols in exercise-mediated inflammation.

## Figures and Tables

**Table 1:** Acute effects of cocoa polyphenols on exercise-mediated oxidative stress, inflammation, and exercise performance.

First Author, Year, (Reference)	Study Design	Number of Participants, Sex, Age, Weight	Exercise Protocol	Interventions (mg Polyphenols Daily)	Measurements	Circulating Leucocyte	IL-6	CK; LDH	Oxidative Stress Markers	EP and R Improvement (Yes/No)
Davidson et al. [58]	RCT, crossover design	14, male, 22 ± 1 years, 71.6 ± 1.6 kg	2.5 h cycling	100 g DCHO (39.1 mg catechin, 96.8 mg epicatechin); cocoa-liquor-free control bar (0 mg); 2 h before exercise	Rest, pre-exercise, post-exercise, after 1 h recovery	↔ by DCHO	↔ in DCHO	NE	TAS: ↑ in DCHO; F2-isoprostane: ↓ by DCHO	No
Decroix et al. [54]	RCT, crossover design	12, male, 30.5 ± 3 years, 72.8 ± 7.8 kg	2 cycling sessions of 30 min;	HFCD (900 mg) or LFCD (15 mg) before first exercise session	Baseline, after 100 and 230 min from supplementation; after training sessions	NE	↔ by CF	NE	TEAC: ↑ by HFCD; MDA: ↔ by HFCD	No
Wiswedel et al. [57]	RCT, double-blind, cross-over	10, male, 20–40 years	Cycling, 29 min	HFCD (187 mg flavanols) or LFCD (14 mg flavanols) 2 h pre-exercise	At baseline and after 2, 4 and 6 h from ingestion	NE	NE	NE	F2-isoprostanes: ↔ by HFCD; ↑ by LFCD	NE
Peschek et al. [56]	RCT, single-blind, cross-over	8, male, 24.6 ± 5.6 years, 73.4 ± 7.0 kg	30 min downhill running; after 48 h, 5 km running	HFCD (2 × 350 mg) or placebo (0 mg) after downhill running	At baseline, after 24 h, after 48 h, after 5 km running	NE	NE	CK and LDH: ↔ by HFCD	NE	No
Stellingwerff et al. [51]	Randomized, single blind, crossover	9, male, 30.0 ± 6.1 years, 72.8 ± 6.0 kg	2.5 h cycling, plus a time trial of 15 min	HFCHO (262 mg) or LFCHO (<0.05 mg) acutely (2 h pre-exercise)	After time trial	NE	NE	NE	NE	No

Abbreviations: CK, creatine kinase; DCHO, dark chocolate; EP, exercise performance; HFCD, high-flavanol cocoa drink; HFCHO, high-flavanol dark chocolate; IL-6, interleukin 6; LDH, lactate dehydrogenase; LFCD, low-flavanol cocoa drink; LFCHO, low-flavanol chocolate; MDA, malondialdehyde; NE, not evaluated; R, recovery; RCT, randomized controlled trial; TAS, Total Antioxidant Status; TEAC, Trolox Equivalent Antioxidant Capacity; ↓, decrease; ↑ increase; ↔ no change.

**Table 2:** Sub-chronic and chronic effects of cocoa polyphenols on exercise-mediated oxidative stress, inflammation, and exercise performance.

First Author, Year, (Reference)	Study Design	Number of Participants, Sex, Age, Weight	Exercise Protocol	Interventions, (Polyphenols Daily mg)	Measurements	Circulating Leucocyte	IL-6	CK; LDH	Oxidative Stress Markers	EP and R Improvement (Yes/No)
Allgrove et al. [47]	RCT, crossover design	20, male, 22 ± 4 years, 74.6 ± 8 kg	Cycling for 90 min followed by 25 min time trial	80 g of DCHO (197.4 mg) or 56.8 g of cocoa liquor-free chocolate control (0 mg) bar for 2 weeks before the trial and on the trial day	Before exercise, post-exercise bout, post- exhaustion, and after 1 h of resting recovery	↔ by DCHO	↔ by DCHO	NE	TAS: ↔ by DCHO; F2-isoprostanes: ↔ in DCHO; ↑ in control	No
Singh et al. [50]	RCT, double-blind, cross-over	16, male, 23 ± 5 years, 79 ± 11 kg	Cycling, 60 min	Cocoa polyphenol supplement (240 mg) or placebo (0 mg) for 7 days	At baseline and after 8 days	NE	NE	NE	TAS: ↔ by cocoa polyphenol supplement	NE
Patel et al. [49]	Randomized, single blind, cross-over	9, male, 21 ± 1 years, 76 ± 9.3 kg	Cycling, 20 min plus a 2 min time trial	40 g DCHO (259 mg) or 40 g white chocolate, 2 weeks	At baseline, after 14 days	NE	NE	NE	NE	Yes
Fraga et al. [48]	RCT, crossover design	28, male, 18 ± 1 years, 74.0 ± 0.2 kg	Football, 2 times a week training	FCMCHO (168 mg) or cocoa butter white chocolate for 2 weeks	At baseline and after 2 weeks	NE	NE	CK:↔ by FCMCHO; LDH: ↓ FCMCHO	MDA: ↓ by FCMCHO; OXOd and TRAP: ↔ by FCMCHO	Yes
Gonzalez-Garrido et al. [55]	Intervention study with pre/post-design	15, male, 17.0 ± 1.11 years, 66.98 ± 6.52 kg	Football training five days/week, and 90 min match/week	HFCD (1050 mg) for 5 days	At baseline and after 6 days	NE	NE	CK and LDH: ↓ by HFCD	TBARS: ↔ by HFCD; MDA: ↓ by HFCD; 4-HNE: ↓ by HFCD; Carbonyl groups: ↓ by HFCD; TAS: ↑ by HFCD	Yes
de Carvalho et al. [53]	RCT, double-blind	13, male, 20.69 ± 1.49 years, 87.02 ± 8.03 kg	Daily rugby match for 5 days	HFCD (2 × 308 mg) or LFCD for 5 days	At baseline and after 6 days	NE	NE	CK: ↔ by HFCD	F2-isoprostanes: ↔ by HFCD	No
Cavarretta et al. [46]	RCT, double-blind	20, male, 17.8 ± 0.9 years (control); 17.4 ± 0.5 years (intervention)	120 min football training, 6 times/week, and 90 min match/week	Normal diet plus 40 g DCHO in tablet (36 mg) or normal diet for 30 days	At baseline and after 60 days	NE	NE	CK and LDH: ↓ by DCHO	HBA: ↓ by DCHO	No
Taub et al. [52]	RCT, double-blind, cross-over	17, male, 49.5 ± 1.6 years, 79 ± 11 kg	Cycling	HFCHO (175 mg) or LFCHO (1.2 mg) for 3 months	At baseline and after 3 months	NE	NE	NE	GSH/GSSG: ↑ by HFCHO; Protein carbonyl: ↓ by HFCHO	Yes

Abbreviations: 4-HNE, 4-hydroxynonenal; CK, creatine kinase; DCHO, dark chocolate; EP, exercise performance; FCMCHO, flavanol-containing milk chocolate; GSH/GSSG, reduced glutathione(GSH) to oxidized glutathione ratio; HBA, hydrogen peroxide (H2O2) breakdown activity; HFCD, high-flavanol cocoa drink; HFCHO, high-flavanol dark chocolate; IL-6, interleukin-6; LDH, lactate dehydrogenase; LFCD, low-flavanol cocoa drink; LFCHO, low-flavanol chocolate; MDA, malondialdehyde; NE, not evaluated; OXOdg, 8-oxo-20-deoxyguanosine; R, recovery; TAS, Total Antioxidant Status; TBARS, thiobarbituric acid reactive substance; TRAP, total relative antioxidant potency; ↓, decrease; ↑ increase; ↔ no change.
